# Brain structure, function, and neurochemistry in schizophrenia and bipolar disorder—a systematic review of the magnetic resonance neuroimaging literature

**DOI:** 10.1038/s41537-017-0013-9

**Published:** 2017-04-03

**Authors:** Badari Birur, Nina Vanessa Kraguljac, Richard C. Shelton, Adrienne Carol Lahti

**Affiliations:** Department of Psychiatry and Behavioral Neurobiology, University of Alabama at Birmingham, Birmingham, AL USA

## Abstract

Since Emil Kraepelin’s conceptualization of endogenous psychoses as dementia praecox and manic depression, the separation between primary psychotic disorders and primary affective disorders has been much debated. We conducted a systematic review of case–control studies contrasting magnetic resonance imaging studies in schizophrenia and bipolar disorder. A literature search in PubMed of studies published between January 2005 and December 2016 was conducted, and 50 structural, 29 functional, 7 magnetic resonance spectroscopy, and 8 combined imaging and genetic studies were deemed eligible for systematic review. Structural neuroimaging studies suggest white matter integrity deficits that are consistent across the illnesses, while gray matter reductions appear more widespread in schizophrenia compared to bipolar disorder. Spectroscopy studies in cortical gray matter report evidence of decreased neuronal integrity in both disorders. Functional neuroimaging studies typically report similar functional architecture of brain networks in healthy controls and patients across the psychosis spectrum, but find differential extent of alterations in task related activation and resting state connectivity between illnesses. The very limited imaging-genetic literature suggests a relationship between psychosis risk genes and brain structure, and possible gene by diagnosis interaction effects on functional imaging markers. While the existing literature suggests some shared and some distinct neural markers in schizophrenia and bipolar disorder, it will be imperative to conduct large, well designed, multi-modal neuroimaging studies in medication-naïve first episode patients that will be followed longitudinally over the course of their illness in an effort to advance our understanding of disease mechanisms.

## Introduction

Since Emil Kraepelin’s conceptualization of endogenous psychoses as two categories, dementia praecox and manic depression, the separation between primary psychotic disorders and primary affective disorders has been much debated.^1–4^ There is substantial evidence that these illnesses share genetic risk factors and overlap in clinical presentation and treatments, suggesting these clinical phenotypes to be on a disease continuum or even differential expressions of the same illness.^5^ Contemporary models attempt to move towards classification of psychiatric disorders based on etiologic and pathophysiologic processes, integrating complex relationships between genetics, physiology, and behavior.^6, 7^

Since the initial description of the principles of magnetic resonance imaging (MRI) by Paul Lauterbur in 1973^8^ and the first human MRI scan in 1977,^9^ much progress has been made in the field of neuroimaging. A variety of contemporary non-invasive sequences are now available to aid structural, functional, and neurochemical characterization of the human brain, providing new opportunities to shed light on biological and pathological processes in vivo.^10^

Conventional, T1-weighted, structural MRI images provide static anatomical information with excellent detail and strong gray/white matter contrast. Manual tracing methods examine brain morphology of pre-defined regions of interest (ROI) such as the hippocampus, but are quite labor intensive. Computational advances offer semi-automated and automated alternatives to manual tracings. One of the most popular automated techniques is voxel-based morphometry (VBM^11^), which quantifies brain structure at the voxel level, with a typical resolution of 1 mm^3^. Another frequently used automated structural imaging analysis technique is Free Surfer,^12^ which also examines cortical thickness, cortical gyrification, and shapes of sub-cortical structures. Diffusion weighted imaging techniques, such as diffusion tensor imaging (DTI), map three dimensional motion of water as a function of spatial location to describe anatomy of anisotropic tissues such as white mater.^13^ The most widely reported diffusion tensor measures are fractional anisotropy (FA) and mean diffusivity (MD) which describe complementary information on structural white matter integrity. Tract based spatial statistics (TBSS) is an automated method describing diffusion metrics from white matter in the entire brain,^14^ whereas fiber tracking quantitatively assess the microstructure of a specific white matter tract.^15^ Fiber reconstruction methods are either deterministic or probabilistic; deterministic methods follow the primary eigenvector from voxel to voxel in three dimensions, whereas probabilistic methods incorporate expected uncertainty into the algorithm.^16^

Functional MRI (fMRI) imaging provides dynamic physiological information by measuring the blood oxygen level dependent (BOLD) signal.^17^ Task fMRI characterizes brain activity while subjects perform cognitive tasks by measuring changes in the BOLD signal in different areas of the brain. In block designs different conditions are alternated in blocks, where the condition assessing the cognitive process of interest is alternated with a control condition, which makes in the change in the fMRI signal in response to the stimulus additive.^18^ Block designs offer considerable statistical power, but are prone to signal drift and run the risk of having subjects become aware of the order of events. On the other hand, event related designs are more reflective of the real world, but come at the expense of statistical power.^19^ Alternatively, resting state fMRI measures the temporal covariance of low frequency fluctuations of the BOLD signal across spatially disparate areas while no explicit task is performed, in an effort to assess the brain’s intrinsic functional organization.^20^ Seed based analysis is a popular hypothesis-driven method to visualize resting state networks.^21^ In this case, an area of interest is defined from which correlations in BOLD fluctuations with all other voxels in the brain are calculated, allowing to examine connectivity of a specific ROI. In contrast, independent component analysis (ICA) is a data-driven method that decomposes the multivariate signal across the brain into statistically independent components (either spatially or temporally) reflecting resting state networks.^22, 23^

Magnetic resonance spectroscopy (MRS) measures chemical composition of tissues, energy metabolism, and neurotransmitter levels in vivo.^24^ The most common type of spectroscopy leverages the signal produced by protons located in the molecules of living tissue to quantify different metabolites (^1^H-MRS). Metabolites that can be measured include N-acetyl-aspartate (NAA), a putative marker of neuronal integrity, choline (Cho), a marker of cellular turnover and cell membrane breakdown, creatine (Cr), a signal that is related to phosphate metabolism, and the amino acids glutamate (Glu), glutamine (Gln), often expressed as Glx (glutamate + glutamine), and ɣ-amino-butyric acid (GABA).^25^ Other spectroscopy techniques include ^31^Phosphorus spectroscopy, which provides a wide range of information on energy metabolism, and ^13^Carbon spectroscopy which use cerebral glucose metabolism to assess glutamine synthesis and glutamatergic transmission.

These complementary imaging techniques have been applied to better delineate the neurobiology of psychiatric disorders in vivo. Structural gray and white matter deficits, as well as neurometabolite alterations, and BOLD signal abnormalities during task and at rest have been found in schizophrenia and bipolar disorder (BD). However, it remains unclear to what extent neural signatures are converging or distinct.

Here, we conducted a systematic review of case–control studies contrasting structural, functional, and neurochemical abnormalities in BD, schizoaffective disorder, schizophrenia, and healthy controls conducted in the past 10 years with the objective to summarize progress made in the quest to better delineate pathophysiological patterns across the psychosis spectrum. Where relevant, selected older publications considered key developments within the field and meta-analyses using quantitative methods to synthesize the literature are also included.

## Results

### Study identification

**Fig. 1:**
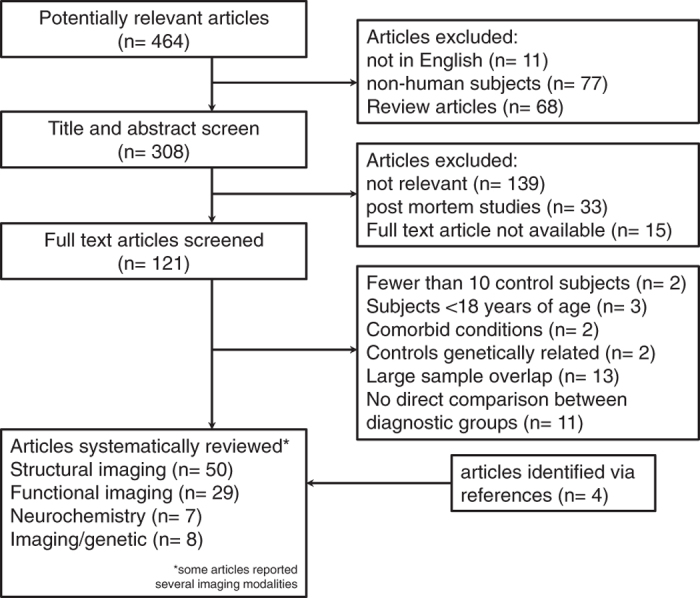
Process of study selection

Figure 1 describes outcomes at each level of our study identification process. Of the 394 potentially relevant articles, we included 50 structural, 29 functional, seven MRS, and eight combined imaging and genetic studies in this systematic review.

### Structural MRI studies

#### Gray matter structural MRI

Gray matter has been an early focus of neuroimaging research in psychiatric illnesses. Using anatomical likelihood estimation (ALE), a meta-analysis of 37 studies demonstrated extensive gray matter deficits in frontal, limbic and subcortical deficits in schizophrenia when compared to healthy controls.^26^ Similarly gray matter reductions were found in anterior cingulate and insula in an ALE meta- analysis of patients with BD when compared to healthy controls.^27^ A recent analyses attempting to quantitatively, albeit not directly, contrast gray matter deficits in a number of psychiatric disorders, including schizophrenia and BD, using meta-analytic techniques found shared decrease in the dorsal anterior cingulate and anterior insula, areas of the brain that are attributed to the salience networks.^28^

Forty studies explicitly examined differences in gray matter across the psychosis spectrum since 2005, but only seventeen of those were conducted at magnetic field strengths of 3T. All but one study included subjects who were medicated at the time of scanning, and only a minority of studies focused on first episode patients (Table 1). Cortical gray matter volume loss appears widespread in schizophrenia, but less extensive^29–31^ or even absent^32, 33^ in BD. It has been suggested as an intermediate phenotype across disease categories, possibly reflecting lifetime psychosis burden, with patients diagnosed with schizophrenia and schizoaffective disorder demonstrating extensive neocortical and subcortical gray matter reductions, and smaller reductions limited to frontotemporal regions in BD.^29^ Further supporting this concept is a recent study by Song et al. who examined gray matter volumes in unmedicated patients with schizophrenia and BD and reported a negative correlation between severity of delusions and frontal gray matter volumes as well as extent of hallucinations and right uncus gray matter volume across diagnostic groups.^34^ Examining subcortical areas of the brain, studies consistently suggest that hippocampal volume reduction may be a feature that is shared across the psychosis spectrum,^35–37^, with a majority of studies reporting that volume loss is greater in schizophrenia compared to BD,^38^ even when examining individual hippocampal subfields.^35, 39^ Similarly, early studies suggest thalamus volume reductions to be present across disease categories,^36, 40^ but later reports with larger sample sizes detected this feature only in schizophrenia.^29, 41^ Findings are more inconsistent in regards to amygdala volume, which has been reported to be unaffected in both diagnostic groups,^42, 43^ decreased only in schizophrenia,^44^ more prominently decreased in schizophrenia compared than in BD^45^ and vice versa.^37^ Reports on basal ganglia volumes are also conflicting where some find volume increase^46, 47^ or decrease^48, 49^ that is shared across illnesses, abnormalities in schizophrenia but not BD,^34, 36^ or lack of abnormalities in either diagnostic group (except for the nucleus accumbens).^50^ Those contrasting reports likely reflect the heterogeneity across studies in regards to patient characteristics, medication exposure, data acquisition, and data analysis methods.

**Table 1:** Studies examining gray matter

Author	Year	n (HC/SZ/SAD/BD)	Bipolar subtype	Illness duration	Medication status	Areas of interest	Analysis method	Tesla	Main findings
Farrow *et al.* ^113^	2005	22/25/0/8	P	FE	medicated	Whole brain	VBM	1.5	HC > SZ in lateral and medial frontal and posterior temporal regions. HC > BD inferior temporal gyri and ACC
Strasser *et al.* ^114^	2005	44/33/0/38	P	Chronic	Medicated	Cerebral volume, hippocampus	MEASURE, ROI (manual tracing)	1.5	HC = BD = SZ in cerebral volume and hippocampus volume
Mc Donald *et al.* ^115^	2006	54/42 SZ/38	P	Chronic	Medicated	Whole brain	MEASURE	1.5	HC = BD = SZ in cerebral volume. HC = BD > SZ in hippocampus
Salisbury *et al.* ^116^	2007	32/20/0/21	P	FE	Medicated	Heschl gyri	ROI (manual tracing)	1.5	SZ < HC = BD in left heschl gyrus gray matter volumes
Nakamura *et al.* ^117^	2007	36/29/0/34	P	FE	Medicated	Neocortex	ROI (manual tracing)	1.5	HC > SZ = BD neocortical gray matter volumes
Morgan *et al.* ^118^	2007	58/44/0/29	P	FE	Medicated	Whole brain	VBM	1.5	No group differences
Frazier *et al.* ^42^	2008	29/20/0/54	P/NP	Chronic	Medicated	Subcortical volumes	ROI (manual tracing)	1.5	No group differences in hippocampus and amygdala volumes
Koo *et al.* ^52^	2008	40/39/0/41	P	FE	Medicated	Cingulate gyrus	ROI (manual tracing)	1.5	HC > SZ in subgenual, anterostratal, anterodorsal, and posterior subregions. HC > BD in the subgenual subregion
Killgore *et al.* ^43^	2009	20/19/0/11		Acute psychosis	Medicated	Cerebrum, amygdala, hippocampus	ROI (manual tracing)	1.5	SZ < BD = HC total brain volume, SCZ = HC = BD in amygdala and hippocampus volume
Reite *et al.* ^119^	2010	89/58/26/51		Chronic	Medicated	Whole brain	manual tracing and semiautomated	1.5/3	No group differences
Molina *et al.* ^48^	2011	24/38/0/19	P/NP	Chronic	Medicated	Whole brain	VBM	1.5	No differences in SZ vs BD. SZ < HC in medial frontal lobe and basal ganglia. BD < HC in medial frontal lobe and caudate
Rimol *et al.* ^33^	2011	207/142/31/139		Chronic	Medicated	Cortical volume	Free Surfer	1.5	SZ < BD in temporal lobe, fusiform and parahippocampal gyrus. BD = HC in cortical volume. Cortical thinning seen in SZ only
Hartberg *et al.* ^36^	2011	192/101/16/121		Chronic	Medicated	Subcortical volumes	Free Surfer	1.5	HC > SZ = BD hippocampus, left thalamus, SZ > BD = HC putamen; HC > BD in cerebellum
Brown *et al.* ^37^	2011	21/17/0/15		Chronic	Medicated	Whole brain, hippocampus, amygdala	VBM	1.5	HC > SZ and BD in frontotemporal regions. HC > SZ > BD in amygdala and right hippocampus
Radonic *et al.* ^120^	2011	15/15/15/15		Chronic	Medicated	Hippocampus	ROI (manual tracing)	2	SZ = SAD < HC = BD in hippocampal volume
de Castro-Manglano *et al.* ^51^	2011	20/10/0/14	P	FE	Medicated	Whole brain	VBM	1.5	HC > BD in frontal lobe, thalamus, superior temporal gyrus, cerebellum; HC > SZ frontal lobe, thalamus, hippocampus; BD > SZ hippocampus
Cui *et al.* ^47^	2011	36/24/0/24	P	Acutely ill FE and chronic	Medicated	Whole brain	VBM	3	HC > SZ in superior temporal and inferior parietal lobe, HC < SZ in putamen; HC > BD in superior temporal, inferior parietal lobe, and caudate; HC < BD in putamen
Ivleva *et al.* ^49^	2012	10/19/16/17	P	Chronic	Medicated	Whole brain	VBM, Free Surfer	3	HC > SZ and SAD in frontal, temporal and insular cortices, HC > SZ across neocortex, thalamus, and basal ganglia, BD = HC in cortical and subcortical gray matter volume
Yuksel *et al.* ^32^	2012	43/37/21/28	P	Chronic	Medicated	Whole brain	VBM	3	HC > SZ in prefrontal and temporal cortices, HC < SZ in cerebellum, BD > SZ in subgenual cortex
Watson *et al.* ^40^	2012	59/25/0/24	P	FE	Medicated	Whole brain	VBM	1.5	HC > SZ in hippocampus, thalamus, striatum and cerebellum. HC > BD in precuneus; BD > HC in cerebellum
Mahon *et al.* ^44^	2012	27/31/0/36	P	Chronic	Medicated	Amygdala	Free Surfer	1.5	Amygdala volume SZ < BD = HC
Ivleva *et al.* ^29^	2013	200/146/90/115	P	Chronic	Medicated	Whole brain	VBM	3	HC > SZ and SAD in frontal, temporal and insular cortices, thalamus, basal ganglia, and cerebellum. HC > BD frontal, insular, temporal and parietal cortex
Ratnanather *et al.* ^121^	2013	27/31/0/36		Chronic	Medicated	STG and planum temporale	ROI	1.5	HC = BD > SZ in planum temporale
Womer *et al.* ^50^	2014	27/28/4/33	P/NP	Chronic	Medicated	Subcortical gray matter	Free Surfer	3	HC = SZ = BP in caudate, putamen, nucleus accumbens, thalamus. BP < HC < SZ in globus pallidus
Haukvik *et al.* ^39^	2014	300/182/28/192		Chronic	Medicated	Hippocampus	Free Surfer	1.5	HC > SZ in all hippocampal subfields, HC > BD in all subfields except for the presubiculum. BD > SZ in presubiculum and subiculum
Mathew *et al.* ^35^	2014	337/219/142/188	P	Chronic	Medicated	Medial temporal lobe	Free Surfer	3	BD = HC > SZ and SAD in medial temporal cortex volume, SZ < BD < HC in hippocampal subfields
Knochel *et al.* ^38^	2014	21/21/0/21		Chronic	Medicated	Hippocampus	VBM	3	Hippocampal volume HC > BD > SZ
Findikili *et al.* ^122^	2015	30/17/0/17		Chronic	Medicated	Pineal gland	ROI (manual tracing)	1.5	Mean pineal gland volume SZ < BD < HC
Nenandic *et al.* ^41^	2015	34/34/0/17	P	Chronic	Medicated	Whole brain	VBM	3	HC > SZ prefrontal cortex and insula, temporal cortex, thalamus, and cerebellum. SZ < BD in hippocampus, DLPFC, and cerebellum
Kittel-Schneider *et al.* ^45^	2015	18/23/0/30	P/NP	FE	Medicated	Amygdala	ROI (manual tracing)	1.5	HC > BD > SZ in amygdala volume
Laidi *et al.* ^123^	2015	52/32/0/115	P/NP	Chronic	Medicated	Cerebellum	Free Surfer	3	SZ < HC = BD in cerebellar volume
Song *et al.* ^34^	2015	35/71/0/44	P	Chronic	Off medication	Whole brain	VBM	3	HC > SZ precentral gyrus, caudate, and cerebellum; HC > BD middle frontal gyrus, fusiform gyrus; BD > SZ cerebellum, temporal lobe, basal ganglia
Pina-Camacho *et al.* ^53^	2015	157/92/0/32/72	P	FE	Medicated	Whole brain	VBM, Free Surfer	1.5/3	HC > SZ in frontal and temporal lobe, SZ > HC in basal ganglia. HC > BD ACC, BD > HC in caudate and temporal lobe thicker temporal cortex
Shepherd *et al.* ^124^	2015	34/28/12/30		Chronic	Medicated	Whole brain	VBM	3	HC > SZ in hippocampus and frontal cortex. HC > BD in precuneus, superior parietal and postcentral gyrus. SZ = BD in GM volume
Amann *et al.* ^125^	2015	45/45/45 /45	P/NP	Mixed	Medicated	Whole brain	VBM	1.5	HC > SZ and SAD in anterior cingulate, insula, temporal lobe, cerebellum; HC = BD
Royer *et al.* ^126^	2015	63/31/0/20		Chronic	Medicated	Whole brain gray matter asymmetry	In house processing	3	No group differences
Poletti *et al.* ^31^	2016	136/96/0/206		Chronic	Medicated	Whole brain	VBM	3	HC > SZ in inferior frontal gyrus, thalamus, insula and superior temporal gyrus; HC > BD inferior frontal gyrus; BD > SZ thalamus
Reavis *et al.* ^127^	2016	30/33/0/31	NP	Chronic	Medicated	Lateral occipital complex and retinotopic visual cortex	ROI	3	HC >BD>SZ in retinotopic cortex and lateral occipital complex
Knoechel *et al.* ^128^	2016	38/32/0/34	NP	Chronic	Medicated	Whole brain	Free Surfer	3	HC > BD = SZ in cortical thickness in inferior frontal gyrus, ACC, PCC. HC > SZ in dorsal frontal and temporal areas. HC > BD in orbitofrontal cortex
Nenadic *et al.* ^129^	2016	38/32/0/34	P	Chronic	Medicated	Whole brain	Free Surfer	3	BD > HC in local gyrification in ACC and DLPFC, SZ > HC in local gyrification in MPFC and orbitofrontal cortex, BD > SZ in ACC gyrification

*HC* healthy control, *BD* bipolar disorder, *SZ* schizophrenia, *SAD* schizoaffective disorder, *P* psychotic bipolar disorder, *NP* non-psychotic bipolar disorder, *FE* first episode, *VBM* voxel based morphometry, *ROI* region of interest analysis, *ACC* anterior cingulate cortex, *PCC* posterior cingulate cortex, *DLPFC* dorsolateral prefrontal cortex, *MPFC* medial prefrontal cortex, *STG superior temporal gyrus*

Significant cortical and subcortical volume loss that resembles the chronic illness stage is already reported in first episode patients. While many of the areas with gray matter loss appear to overlap across diagnostic groups, several reports suggest greater abnormalities in first episode schizophrenia compared to first episode BD, both in terms of volume loss^45, 51^ and spatial extent.^40, 52^ It is perhaps not surprising that Pina-Camacho et al. reported that age at first onset of psychosis modulated structural abnormalities in a nonlinear and diagnosis dependent manner. Specifically, they report that patients with an earlier onset of a schizophrenia spectrum disorder had the most significant ventricular and basal ganglia enlargement along with the greatest fronto-temporal cortical volume and thickness deficits among diagnostic groups, with affective disorder patients having less extensive cortical deficits that were again more prominent in those with younger age of onset of psychosis.^53^ However, none of the studies examined medication naïve patients, making it impossible to definitively conclude that observed group differences in gray matter abnormalities are due to intrinsic differences across the diagnostic spectrum rather than secondary to differential exposure to psychotropic medications.

#### White matter structural MRI

White matter abnormalities have been reported to be widespread in both schizophrenia and BD. An activation likelihood estimation meta-analysis showed decreased FA in first episode schizophrenia compared to healthy controls across the commissural, association, and projection tracts, with main involvement of the inferior fronto-occipital fasciculus, inferior longitudinal fasciculus, cingulum bundle, and corpus callosum.^54^ Similarly, a meta-analysis of fifteen DTI studies in BD reported decreased FA in all types of tracts when compared to healthy controls, with the most robust decreases in the inferior fronto-occipital fasciculus.^55^ Findings suggest a shared spatial distribution of white matter integrity deficits across the illness spectrum, but allow no inference on the comparability in magnitude of abnormalities.

Eleven studies contrasted white matter integrity in schizophrenia and BD; all but one included patients who were medicated at the time of assessment, and only two focused on first episode patients (Table 2). It is striking that the majority of studies conducted in chronic patients on psychotropic medications show decreases in white matter integrity that do not appear different across diagnostic groups. Region of interest analyses show shared FA decreases in the uncinate fasciculus, corona radiata, anterior limb of the internal capsule, anterior and posterior thalamic radiation, and corpus callosum.^56–60^ This is also corroborated by Skudlarski and colleagues who conducted the largest study thus far and found a close agreement between spatial distributions and magnitudes in FA reductions assessed with whole brain TBSS across diagnostic groups. Interestingly, they reported higher variance in patients with psychotic BD, suggestive of greater heterogeneity in white matter integrity abnormalities compared to patients with schizophrenia.^61^ Heterogeneity may also explain discrepancies with Anderson and colleagues who found FA reductions in temporal and occipital white matter in schizophrenia but not bipolar I disorder,^62^ and Knoechel and colleagues who report that the magnitude of white matter integrity abnormalities in the left cingulum and right uncinate fasciculus is greater in schizophrenia than BD.^38^

**Table 2:** Studies examining white matter

Author	Year	n (HC/SZ/ SAD/BD)	Bipolar subtype	Illness duration	Medication status	Area of interest	Tesla	Direct-ions	Analysis type	Main findings
McIntosh	2008	49/25/0/40		Chronic	Medicated	Uncinate fasciculus and anterior thalamic radiation	1.5	51	Tractography	HC > BD and SZ in FA in the uncinate fasciculus and anterior thalamic radiation
Sussmann *et al.* ^59^	2009	38/28/0/42		Chronic	Medicated	Uncinate fasciculus and internal capsule	1.5	51	ROI analyses	HC > BD = SZ in FA in internal capsule, HC > BD in anterior limb of internal capsule
Cui *et al.* ^130^	2011	30/25/0/18	P	Chronic	Medicated	Whole brain	3	15	Voxel based analysis	HC > SZ = BD in FA in posterior corona radiata, HC > BD in FA in fronto-parietal white matter
Lu *et al.* ^64^	2011	18/21/0/13	P	FE	Minimally treated/ medication naïve	Whole brain	3	27	VBM	HC > BP = SZ in MD in cingulum, corpus callosum, corona radiata, internal capsule, and occipital WM including posterior thalamic radiation, inferior longitudinal fasciculus/inferior fronto-occipital fasciculus. HC = SZ > BD in FA in multiple commissural, projection, and association tracts
Sui *et al.* ^57^	2011	62/54/0/48		Chronic	Medicated	Whole brain	3	12	Tractography	HC > BD and SZ in FA in occipital and frontal lobes
da Cunha Colombo *et al.* ^63^	2012	94/55/7/26	P	FE	Medicated	Whole brain	1.5		VBM	HC = SZ = BD in regional WM volume
Anderson *et al.* ^62^	2013	56/35/0/20	P/NP	Chronic	Medicated	21 regions	1.5	25	ROI analyses	HC = BD > SZ in FA in superior temporal, parahippocampal, and occipital white matter; HC < BD = SZ in MD in superior temporal, parahippocampal, inferior frontal, fusiform, angular white matter; BD > SZ in MD precentral and middle frontal cortex
Skudlarski *et al.* ^61^	2013	104/109/35/63	P	Chronic	Medicated	Whole brain	3	32	TBSS	HC > SZ = BD in FA in 29 of 76 regions analyzed
Knochel *et al.* ^38^	2014	21/21/0/21		Chronic	Medicated	Uncinate fasciculus, cingulum, fornix	3	60	TBSS	HC = BD < SZ in MD in the cingulum; HC > SZ = BD in MD in the fornix
Li *et al.* ^60^	2014	24/19/0/16		Chronic	Medicated	Corpus Callosum	3	25	ROI analyses	HC > SZ in entire corpus callosum, HC > BD in all subregions except the middle genu
Kumar *et al.* ^56^	2015	41/34/6/22	P	Chronic	Medicated	Whole brain	3	32	TBSS/ Tractography	HC > SZ = BD in FA in corpus callosum, corona radiata

*HC* healthy control, *BD* bipolar disorder, *SZ* schizophrenia, *SAD* schizoaffective disorder, *P* psychotic bipolar disorder, *NP* non-psychotic bipolar disorder, *FE* first episode, *TBSS* tract based spatial statistics, *ROI* region of interest, *FA* fractional anisotropy, *MD* mean diffusivity, *WM* white matter

In first episode patients, no white matter volume abnormalities or no differences across diagnostic groups were observed^63^. The only study to date examining white matter microstructure in medication-naïve and minimally treated patients found a shared increase in MD in a large number of white matter tracts across diagnostic categories, but showed that patients with first episode BD had decreased FA in the cingulum, internal capsule, and posterior brain regions that was not evident in first episode schizophrenia.^64^

### Functional MRI studies

#### Task functional MRI

A total of thirteen studies, all in chronically medicated patients, have directly contrasted functional activation patterns in BD and schizophrenia using various tasks (Table 3). In working memory tasks, studies largely observe engagement of the same brain networks in healthy controls and patients across the psychosis spectrum, but find alterations in activation patterns within those networks. A graded pattern of group differences in the amplitude of the BOLD signal has been reported in several studies, with the greatest alteration typically reported in schizophrenia, and more subtle or lack of abnormalities in BD.^65–67^ Findings are more inconsistent, with both hypo- and hyper-activation reported, which may be explained by different, but overlapping, inverted u-shaped curves of activation depending on task difficulty^68^ across the psychosis spectrum, with less abnormal patterns of activation in BD compared to schizophrenia. Alternatively, it is possible that these patterns of differential activation could represent compensatory processes or secondary effects of primary changes in signal processing. Greater task related alterations in schizophrenia compared to BD were also reported in a verbal fluency task,^69^ but not a sentence completion task,^70^ or emotionally salient memory tasks,^71, 72^ the latter appearing more altered in BD than schizophrenia. Furthermore, activation during reward anticipation in the ventral striatum appears decreased in schizophrenia, but not patients with BD in a manic state.^73^ The authors speculated that striatal dopamine dysfunction, which could be clinically expressed as anhedonia, may be underlying their finding.

**Table 3:** Studies examining task activation

Author	Year	n (HC/SZ/SAD/BD)	Bipolar subtype	Illness duration	Medication status	Tesla	Task	Design	Main findings
McIntosh *et al.* ^70^	2008	37/27/0/42		Chronic	Medicated	1.5	Hayling sentence completion test	Block	BD > HC activation in VLPFC, striatum and caudate. SZ < HC in the DLPFC
Hamilton *et al.* ^131^	2009	38/20/0/21		Chronic	Medicated	3	Visual working memory	Block	HC > SZ in activation in inferior frontal gyrus and DLPFC. HC > BD in activation in occipital regions, SZ > BD in precentral and postcentral gyrus, medial frontal gyrus and parietal regions
Costrafreda *et al.* ^132^	2009	48/39/0/28		Chronic	Medicated	1.5	Phonological letter fluency	Block	SZ > HC = BD in activation in inferior frontal cortex
Whalley *et al.* ^71^	2009	14/15/0/14		Chronic	Medicated	1.5	Emotionally salient memory	Block	BD > HC > SZ in left hippocampus, BD > HC in posterior cingulate, STG and precentral gyrus. BD > SZ in posterior cingulate, superior temporal sulcus, precentral gyrus. SZ < HC in amygdala
Hall *et al.* ^133^	2009	14/15/0/14		Chronic	Medicated	1.5	Face/name encode and retrieve	Block	HC = BD > SZ in activation of anterior right hippocampus during encode, BD < SZ = HC in activation of left DLPFC during encode. SZ > BD in DMPFC activation during retrieval, SZ > HC in DLPFC activation during retrieval
Milanovic *et al.* ^66^	2011	19/10/0/12	P	Chronic	Medicated	1.5	n-Back	Block	SZ > HC = BD activation in the MPFC region of interest, but no group differences in the DLPFC region of interest
Costrafreda *et al.* ^69^	2011	40/32/0/32		Chronic	Medicated	1.5	Phonological letter fluency	Block	SZ > BD > HC activation in anterior cingulate, middle frontal gyrus, and putamen. SZ > BD = HC superior, middle, and inferior frontal gyrus; SZ = BD > HC in precuneus, posterior cingulate, and angular gyrus
Morris *et al.* ^72^	2012	15/12/0/13		Chronic	Medicated	3	Emotionally salient memory	Block	BD > SZ > HC in emotion downregulation in the prefrontal cortex; BD = SZ > HC in emotion upregulation in prefrontal cortex
Palaniyappan *et al.* ^134^	2013	34/34/0/20	P	Chronic	Medicated	3	n-Back	Block	HC > SZ in degree centrality in frontal lobe, STG and insula, HC > BD in degree centrality in insula, SZ > HC in hippocampus, thalamus, and fusiform gyrus, BD > HC hippocampus, thalamus, and caudate
Brandt *et al.* ^65^	2014	100/86/14/100	P/NP	Chronic	Medicated	1.5	n-Back	Block	SZ > BD > HC in activation in the dorsolateral and ventrolateral PFC, premotor cortex and parietal cortex; HC > BD > SZ in inferior frontal gyrus, inferior parietal cortex, STG, and precuneus
Wu *et al.* ^67^	2014	29/36/0/20		Chronic	Medicated	3	n-Back	Block	SZ > BD > HC in hyperactivity in the PCC and MPFC
Zhang *et al.* ^135^	2015	21/17/0/17	P	Chronic	Medicated	3	self-reflection task	Block	HC > BD in PCC precuneus activation during other vs. self-contrast
Hagele *et al.* ^73^	2015	54/44/0/13		Chronic	Medicated	1.5	Monetary incentive delay		SZ showed less ventral striatal activation compared to HC = BD during reward anticipation. No group differences in loss anticipation

*HC* healthy control, *BD* bipolar disorder, *SZ* schizophrenia, *SAD* schizoaffective disorder, *P* psychotic bipolar disorder, *NP* non-psychotic bipolar disorder, *PFC* prefrontal cortex, *DLPFC* dorsolateral prefrontal cortex, *DMPFC* dorsomedial prefrontal cortex, *MPFC* medial prefrontal cortex, *VLPFC* ventrolateral prefrontal cortex, *STG* superior temporal gyrus, *PCC* posterior cingulate cortex

#### Resting state functional MRI

Investigation of functional connectivity at rest has become increasingly popular, in part because task performance differences between groups need not be accounted for in this paradigm.^74^ An activation likelihood estimation meta-analysis of whole brain resting state studies in schizophrenia suggests decreased activity in the medial prefrontal cortex, left hippocampus, posterior cingulate cortex and precuneus (all areas of the brain that are typically conceptualized as part of the default mode network^75^), as well as increased activity in the lingual gyrus.^76^ A recent review attempting to reconcile methodological differences in schizophrenia studies suggested increased functional connectivity to be a replicated finding.^77^ In a qualitative systematic review Vargas and colleagues reported aberrant resting state connectivity in between frontal and meso-limibic areas in BD when compared to controls.^78^

All of the sixteen resting state studies comparing connectivity across diagnostic groups included here were conducted in patients who were medicated at the time of scanning (Table 4). The default mode network, a large scale brain network that is more active at rest and has been implicated in self-referential thinking, is perhaps the most widely studied. An early report in acutely ill patients with schizophrenia and BD identified the medial prefrontal cortex as major locus of shared abnormality, with BD being characterized by reduced default mode network connectivity to the hippocampus and fusiform gyrus as well as increased connectivity with the primary visual cortex, and schizophrenia being characterized by abnormal recruitment of the frontal polar cortex and the basal ganglia.^79^ The largest study to date examining default mode network connectivity with ICA reported connectivity reductions in the medial prefrontal cortex, anterior cingulate cortex, posterior cingulate cortex and precuneus across the psychosis spectrum, but also found that selective nodes within the network appear to be differentially affected in schizophrenia and BD.^80^ The same group also reported aberrant connectivity between the default mode and a fronto-occipital network as shared illness feature in schizophrenia and BD, whereas increased connectivity between fronto-temporal and mesolimbic regions was only evident in BD, and decreased connectivity between sensory-motor and mesolimbic areas was limited to schizophrenia.^81^ Others report within and between network connectivity decreases within a cingulo-opercular network and between a cingulo-opercular and cerebellar network that are shared across illnesses, decreased connectivity between the cingulo-opercular and salience network in BD only, and decreased connectivity between cingulo-opercular and fronto-parietal network in schizophrenia only. Notably, default mode network connectivity was not reported abnormal.^82^ Similarly, Baker and colleagues reported resting state connectivity disruptions of cortical association networks, preferentially the frontoparietal control network, but not default mode network abnormalities, in schizophrenia and BD.^83^

**Table 4:** Resting state studies

Author	Year	n (HC/SZ/SAD/BD)	Bipolar subtype	Illness duration	Medication status	Areas of interest	Tesla	Scan (min)	eyes open/closed	Analysis	Main findings
Ongur *et al.* ^79^	2010	15/7/7/17		Acutely ill	Medicated	Default mode network	3	10	Open	ICA	Mixed increase and decrease in DMN connectivity in both SZ and BD compared to HC
Chai *et al.* ^136^	2011	15/16/0/14		Chronic	Medicated	Medial prefrontal cortex	3	10	Open	Seed	BD > SZ > HC in ventrolateral prefrontal cortex and insula connectivity; HC > SZ > BD in DLPFC connectivity
Meda *et al.* ^81^	2012	118/70/0/64	P	Chronic	Medicated	16 Large scale networks	3	5.25	Open	ICA	HC > SZ = BD in fronto-occipital and anterior DMN, HC = BD > SZ in meso/paralimbic and sensory-motor network, BD > HC in Fronto-temporal/paralimbic and meso/paralimbic
Mamah *et al.* ^82^	2013	33/25/0/35		Chronic	Medicated	Five large scale networks	3	13.7	Closed	Seed	HC > BD = SZ in cingulo-opercular network
Liu *et al.* ^85^	2014	18/18/0/18		Chronic	Medicated	Prefrontal cortex, amygdala	3		Closed	Seed	BD = SZ mixed increase and decrease in connectivity between amygdala subregions and the prefrontal cortex compared to HC
Meda *et al.* ^80^	2014	324/269/0/300	P	Chronic	Medicated	Default mode network				ICA	HC > BD > SZ
Anticevic *et al.* ^84^	2014	146/90/0/73	P/NP	Chronic	Medicated	Thalamus	3	5.25	Open	Seed	Mixed increase and decrease in connectivity in SZ and BD compared to HC; BD show less cerebellar dysconnectivity than SZ
Knochel *et al.* ^38^	2014	21/21/0/21		Chronic	Medicated	Hippocampus	3	13.3		Seed	No significant differences when all three groups are compared
Rashid *et al.* ^137^	2014	61/60(SZ/ SAD)/0/38	P/NP	Chronic	Medicated	49 Large scale networks	3	5.25	Open	Dynamic ICA	HC > BD = SZ in number of transitions between states
Argyelan *et al.* ^87^	2014	32/18/0/19	P/NP	Chronic	Medicated	Whole brain connectivity, caudate	3	5	Closed	Seed	SZ < BD < HC in global connectivity, specifically thalamus, paracingulate, caudate, fusiform and lingual gyrus
Baker *et al.*JT^83^	2014	100/28/32/40	P	Chronic	Medicated	17 Large scale networks	3	6.2	Open	Seed	HC > BD = SZ in frontoparietal network
Meda *et al.* ^138^	2015	242/220/147/180	P	Chronic	Medicated	Whole brain	3	5	Open	ALFF	Mixed increase and decrease in ALFF in all diagnostic groups
Du *et al.* ^139^	2015	20/20/33/20		Chronic	Medicated	12 Large scale networks	3	5	Open	ICA	Discriminative regions included frontal, parietal, precuneus, cingulate, supplementary motor, cerebellar, insula and supramarginal cortices
Anticevic *et al.* ^140^	2015	56/73/0/73	P/NP	Chronic	Medicated	Ventral ACC	3	5.25	Open	Seed	HC > SZ = BD
Hager *et al.* ^141^	2016	156/107/98/125	P	Chronic	Medicated	Whole brain	3	5		Multiple time scales	SZ < HC in complexity in hypothalamus, BD < HC in complexity in left inferior occipital, right precentral and left superior parietal. HC > SZ = SAD = in complexity PFC
Skatun *et al.* ^142^	2016	196/60/11/43		Chronic	Medicated		3	5.3	Open	Eigenvector centrality mapping	HC > SZ in global connectivity in sensory regions, HC > SZ = BD in subcortical regions; SZ > BD > HC in connectivity in frontal and parietal areas

*HC* healthy control, *BD* bipolar disorder, *SZ* schizophrenia, *SAD* schizoaffective disorder, *P* psychotic bipolar disorder, *NP* non-psychotic bipolar disorder, *ICA* independent components analysis, *ALFF* amplitude of low frequency fluctuations, *ACC* anterior cingulate cortex, *PFC* prefrontal cortex, *DLPFC* dorsolateral prefrontal cortex, *DMN* default mode network

Resting state studies of non-neocortical structures revealed distinct patterns of thalamic^84^ and amygdalar^85^ dysconnectivity in schizophrenia and BD, but no differences across diagnostic groups in hippocampal^38, 86^ connectivity. Examining connectivity across the entire brain with global brain connectivity, a measure that computes connectivity strength of every region of the brain with every other region of the brain, was reported to be lower in schizophrenia than in healthy controls, whereas patients with BD had intermediate global connectivity strength that was significantly different from both patients with schizophrenia and healthy controls.^87^

### Magnetic resonance spectroscopy studies

There is substantial evidence that neurometabolite levels are altered in both schizophrenia and BD. A meta-analysis pooling data from 146 studies suggests decreases in NAA in the frontal lobe, hippocampus, thalamus, and basal ganglia in schizophrenia, but only in the basal ganglia and frontal lobe in BD.^88^ Another meta-analysis summarizing findings of glutamatergic abnormalities across 28 studies in schizophrenia revealed a decrease in medial frontal glutamate compared with healthy controls,^89^ but the majority of studies were conducted in medicated patients. Contrastingly, several reports do suggest an elevation of glutamatergic indices in unmedicated patients with schizophrenia in the medial prefrontal cortex, striatum, and hippocampus.^90–93^ A smaller meta-analysis in BD including nine studies measuring Glx (a combination of glutamate and glutamine) across different areas of the brain, suggested that this metabolite may be higher in patients with BD compared to controls, irrespective of medication status.^94^ Taken together, it appears that some of the neurometabolite alterations, specifically decreased NAA in the frontal cortex and basal ganglia may be shared across the illness spectrum, whereas others may not.

However, studies directly contrasting neurometabolites in BD and schizophrenia are sparse. All of these studies used single voxel^1^ H-MRS placed in cortical areas of the brain and were conducted in chronically ill patients who were medicated at the time of assessment, most commonly with very small sample sizes (Table 5). Molina and colleagues were the first to report that NAA/Cr decreases in the left, but not the right dorsolateral prefrontal cortex were greater in schizophrenia than in BD, with no Cho/Cr abnormalities appreciated in either group.^95^ Findings were partially replicated by Kalayci and colleagues, who reported a universal decrease in left and right dorsolateral prefrontal cortex NAA/Cr across diagnostic groups, but a decrease in Cho/Cr only in BD and schizoaffective disorder, but not schizophrenia when compared to controls.^96^ Anterior cingulate cortex metabolite measurements at 1.5 Tesla were suggestive of elevations of Cho/Cr in schizophrenia only without detectable abnormalities in NAA/Cr across groups.^97^ However, because of a later report of decreased Cr levels in acutely ill patients with schizophrenia but not BD, it is unclear if findings are attributable to Cr alterations rather than Cho or NAA changes.^98^ In the left Heschl’s gyrus, Glu, NAA, and inositol levels were found to be decreased in BD, but not in schizophrenia when compared to healthy controls, while no metabolite abnormalities in either diagnostic group were detected in the right Heschl’s gyrus, suggesting a lateralized abnormality in the dominant hemisphere.^99^ Only one study to date has examined GABA, with reports of perisylvian GABA elevations in patients with schizophrenia, but not those with BD, when compared to healthy controls.^100^

**Table 5:** Studies examining magnetic resonance spectroscopy (MRS)

Author	Year	n (HC/SZ/ SAD/ BD)	Bipolar subtype	Illness duration	Medication status	Area of interest	MRS	Sequence	Tesla	Main findings
Molina *et al.* ^95^	2007	10/11/0/13	P/NP	Chronic	Medicated	DLPFC	SV	PRESS	1.5	NAA/Cr decreased in SZ and BD No abnormalities in Cho/Cr in either group compared to HC
Crespo *et al.* ^97^	2008	15/14/0/17	NP	Chronic	Medicated	dACC	SV	PRESS	1.5	NAA/Cho SZ < HC and BD; Cho/Cr SZ > HC and BD; NAA/Cr no group differences
Ongur *et al.* ^98^	2009	22/8/7/15	P	Chronic	Medicated	ACC/POC	SV	PRESS	4	Cr HC = BD > SZ in both voxels
Ongur *et al.* ^143^	2010	20/15/0/15	P	Chronic	Medicated	ACC/ POC	SV	PRESS	4	Numerically shortened T2 relaxation times in BD and SZ
Kalayci *et al.* ^96^	2012	15/15/15/15		Chronic	Medicated	DLPFC	SV	PRESS	1.5	NAA HC > BD, SZ, SAD; Cho HC > BD, SAD; Cr C > BD, SAD, SZ
Atagun *et al.* ^99^	2015	30/30/0/28		Chronic	Medicated	Heschl’s gyrus	SV	PRESS	3	Glu, NAA, and Cr HC > BD in left hemisphere; no group differences in right hemisphere
Atagun *et al.* ^100^	2016	30/25/0/48		Chronic	Medicated	Perisylvian structures	SV	MEGA-PRESS	3	GABA SZ > BD = HC

*HC* healthy control, *BD*, bipolar disorder, *SZ* schizophrenia, *SAD* schizoaffective disorder, *P* psychotic bipolar disorder, *NP* non-psychotic bipolar disorder, *DLPFC* dorsolateral prefrontal cortex, *ACC* anterior cingulate cortex, *dACC* dorsal anterior cingulate cortex, *POC* parieto-occipital cortex, *SV* single voxel, *PRESS* point resolved spectroscopy sequence, *NAA* N-acetyl-aspartate, *Cr* creatine, *Cho* choline, *Glu* glutamate, *GABA* gamma-aminobutyric acid

### Imaging-genetic studies

Eight studies, all in medicated patients, have examined relationships between imaging and genetic markers across the illness spectrum (Table 6). In verbal fluency tasks, significant diagnosis by genotype interactions with task related activations were observed for Neuregulin 1,^101^ disrupted in Schizophrenia Gene 1 (DISC 1^102^), and the d-amino acid oxidase (See ref. 103) gene. Interestingly, a modest relationship between white matter volume and a number of schizophrenia risk genes was found across the psychosis spectrum and in healthy controls, suggesting that cumulative genetic risks may help explain the extent of observed white matter alterations,^104^ but other risk genes did not show such relationships.^105^

**Table 6:** Imaging genetic studies

Author	Year	n (HC/SZ/SAD/BD)	Bipolar subtype	Illness duration	Medication status	Genes	Imaging modality	Design	Analysis type	Tesla	Main findings
Mechelli *et al.* ^101^	2008	45/41/0/29		Chronic	Medicated	Neuregulin 1	Verbal fluency task fMRI	Block design	Full factorial ANOVA	1.5	HC > BD = SZ in activation in the angular gyrus. Significant diagnosis x genotype interaction in prefrontal cortex activation
Prata *et al.* ^102^	2011	53/44/0/35	P/NP	Chronic	Medicated	DISC 1	Verbal fluency task fMRI	Block design	Full factorial ANOVA	1.5	HC < SZ in activation in middle frontal gyrus, no differences between HC and BD. Group × genotype interaction (HC and SZ) in left superior frontal gyrus activation
Papagni *et al.* ^103^	2011	48/40/0/33		Chronic	Medicated	DAAO	Verbal fluency task fMRI	Block design	Full factorial ANOVA	1.5	HC > SZ in activation of the PCC, no group differences between SZ and BD. Diagnosis x genotype interaction in precuneus and PCC
Tesli *et al.* ^144^	2013	123/52/9/66	P	Chronic	Medicated	CACNAC1C	Emotionally salient fMRI	Block design	Full factorial ANOVA	1.5	No significant diagnosis x genotype interaction in all groups
Kittel-Schneider *et al.* ^45^	2015	18/23/0/30	P/NP	FE	Medicated	DGKH	Structural MRI	ROI (manual tracing)	ANOVA	1.5	HC > BD > SZ in left amygdala volume. Significant gene × volume effect in right amygdala
Oertel-Knochel *et al.* ^104^	2015	50/24/0/20		Chronic	Medicated	7 SZ risk SNPs	Structural MRI	VBM	Hierarchal regression analyses	3	HC > SZ = BD in white matter volume, association between risk genes and white matter volume found only in HC, not SZ or BD
Mallas *et al.* ^105^	2016	124/63/0/42	NP	Chronic	Medicated	CACNAA1C	Diffusion tensor imaging	TBSS	ANOVA	1.5	No genotype main effect, no group by genotype interaction
Tandon *et al.* ^145^	2016	123/139/90/160	P	Chronic	Medicated	Large number of SNPs	Structural MRI	Free surfer	Para-ICA	3	Four structural and three genetic components that showed overlapping relationships with the disease risk genes across illnesses

*HC* healthy control, *BD* bipolar disorder, *SZ* schizophrenia, *SAD* schizoaffective disorder, *P* psychotic bipolar disorder, *NP* non-psychotic bipolar disorder, *FE* first episode, *ROI* region of interest, *TBSS* tract based spatial statistics, *ANOVA* analysis of variance, *ICA* independent components analysis, *PCC* posterior cingulate cortex

## Discussion

In this systematic review, we sought to summarize converging and distinct neural signatures in schizophrenia and BD. Structural neuroimaging studies suggest white matter integrity deficits that are consistent, both in magnitude and spatial extent, across the psychosis spectrum, while gray matter reductions, especially those that are cortical, appear more widespread in schizophrenia compared to BD. Similarly, spectroscopy studies in cortical gray matter report evidence of decreased neuronal integrity in both disorders, but not enough data exists to draw firm conclusions as to differences in magnitudes and spatial distribution between illnesses. On a functional level, findings are inconsistent, possibly because of small sample sizes in many of the studies. Functional MRI studies using task paradigms typically report engagement of the same brain networks in healthy controls and patients across the psychosis spectrum, but find differential extent of alterations in magnitude of task related activation between illnesses depending on the task paradigm. The larger resting state connectivity studies are inconsistent as to abnormalities in the default mode network, but it appears that decreased fronto-parietal network connectivity may be a shared feature across the psychosis spectrum. The very limited imaging-genetic literature suggests a relationship between psychosis risk genes and brain structure, and possible gene by diagnosis interaction effects on functional imaging markers.

Our work needs to be considered in context of several limitations. We performed a systematic review, but did not perform quantitative assessments using meta-analytic techniques which could be informative in future work. We did not do separate analyses in schizophreniform disorder or schizoaffective disorder due to a lack of studies investigating these as a distinct disease category (schizoaffective disorder is most commonly grouped under schizophrenia). We decided not to include ultra-high risk subjects as conversion rates to psychotic illness is reported to be 30% or less,^106^ and psychotic depression due to the paucity of studies including this disease category.

It is important to note that the vast majority of studies have been conducted in chronically ill, medicated patients, which precludes us from being able to disentangle intrinsic illness characteristics from changes attributable to disease progression and exposure to psychotropic medications. While the existing literature suggests some shared and some distinct neural markers in across the psychosis spectrum, it will be imperative to conduct large, well designed, multi-modal neuroimaging studies in medication-naïve first episode patients that will be followed longitudinally over the course of their illness in an effort to advance our understanding of disease mechanisms and to resolve the illness dichotomy vs. illness spectrum debate. But because this type of studies are notoriously difficult to conduct, and sample sizes are typically fewer than 50 subjects,^107–112^ a potential alternative strategy may be to obtain very large, multi-site datasets and attempt to mitigate medication confounds with statistical adjustments.

## Methods

### Eligibility criteria

Studies were included if they presented original data published between January 2005 and December 2016 (last search December 9th 2016), compared individuals with BD and schizophrenia/schizoaffective disorder and healthy controls. Studies were not included when the healthy control group was genetically related to the patient groups. Studies published in languages other than English, post mortem studies, non-human studies, and review articles were excluded. We only included trials with ten or more healthy subjects, aged 18 or older. Studies expressively including subjects with comorbid substance use disorders, neurological or genetic diseases, or intellectual disabilities were not considered. When a single study was published in several articles, the article reporting the largest group was used. Articles that did not explicitly compare imaging findings between diagnostic groups were excluded as well.

### Literature search

BB and NVK performed a literature search in PubMed including subjects across the psychosis spectrum using the following key words: (Schizophrenia AND bipolar disorder) AND (gray matter OR morphometry OR VBM OR volume OR white matter OR DTI OR magnetic resonance spectroscopy OR MRS OR functional MRI OR resting state). The reference lists of included studies, as well as relevant meta-analyses were inspected for additional eligible publications.

### Study selection

After removal of duplicate articles, BB and NVK screened titles and abstracts retrieved from the search and selected potentially eligible studies for full text review. Both authors applied eligibility criteria, and a list of eligible full text articles was developed through consensus. Full text articles were then downloaded or requested from the university library and assessed for eligibility. Figure 1 describes the study selection process and outcome.

### Data extraction

We extracted the following data from each study: name of first author, year of publication, number of participants per diagnostic category, illness duration, mood state, use of psychotropic medications, data acquisition parameters, magnetic field strength, data processing parameters, main study outcomes.
